# Role of Artificial Intelligence in Unruptured Intracranial Aneurysm: An Overview

**DOI:** 10.3389/fneur.2022.784326

**Published:** 2022-02-23

**Authors:** Anurag Marasini, Alisha Shrestha, Subash Phuyal, Osama O. Zaidat, Junaid Siddiq Kalia

**Affiliations:** ^1^AINeuroCare Academy, Dallas, TX, United States; ^2^Travel and Mountain Medicine Center, Kathmandu, Nepal; ^3^Department of Neurointerventional Radiology, Upendra Devkota Memorial National Institute of Neurological and Allied Sciences, Kathmandu, Nepal; ^4^Departments of Endovascular Neurosurgery and Stroke, St. Vincent Mercy Medical Center, Toledo, OH, United States; ^5^Clinical Strategy, VeeOne Health Inc., Roseville, CA, United States; ^6^neurologypocketbook.com, Dallas, TX, United States

**Keywords:** artificial intelligence, machine learning, aneurysm rupture, unruptured intracranial aneurysms (UIAs), deep learning

## Abstract

Intracranial aneurysms (IAs) are a significant public health concern. In populations without comorbidity and a mean age of 50 years, their prevalence is up to 3.2%. An efficient method for identifying subjects at high risk of an IA is warranted to provide adequate radiological screening guidelines and effectively allocate medical resources. Artificial intelligence (AI) has received worldwide attention for its impressive performance in image-based tasks. It can serve as an adjunct to physicians in clinical settings, improving diagnostic accuracy while reducing physicians' workload. AI can perform tasks such as pattern recognition, object identification, and problem resolution with human-like intelligence. Based on the data collected for training, AI can assist in decisions in a semi-autonomous manner. Similarly, AI can identify a likely diagnosis and also, select a suitable treatment based on health records or imaging data without any explicit programming (instruction set). Aneurysm rupture prediction is the holy grail of prediction modeling. AI can significantly improve rupture prediction, saving lives and limbs in the process. Nowadays, deep learning (DL) has shown significant potential in accurately detecting lesions on medical imaging and has reached, or perhaps surpassed, an expert-level of diagnosis. This is the first step to accurately diagnose UIAs with increased computational radiomicis. This will not only allow diagnosis but also suggest a treatment course. In the future, we will see an increasing role of AI in both the diagnosis and management of IAs.

## Introduction

Intracranial aneurysms (IAs) are a significant public health concern. In populations without comorbidity and a mean age of 50 years, their prevalence is up to 3.2% (1). With the development and application of advanced imaging techniques worldwide, unruptured intracranial aneurysms (UIAs) are being detected more frequently. Subarachnoid hemorrhage (SAH) owing to UIA rupture accounts for 5–10% of all strokes in the United States (2) SAH may cause considerably high mortality, and those who survive may endure chronic neuropsychological effects and decreased quality of life (3). Whether detected incidentally or during screening, the management of intracranial aneurysms (IA) is a challenge in itself for both the treating physician and patients. This challenge cannot be overcome easily by conventional methodology because the decision to intervene involves weighing the periprocedural risks innate to endovascular or surgical methods and subarachnoid hemorrhage (4). Due to a lack of clear understanding of the natural history of small UIAs and the heterogeneity in the current guidelines and literature, there is significant variability in the perceptions and surveillance practices for small UIAs (<7 mm). This was discussed in a survey of 227 practicing neuroradiologists and neurointerventionalists. Where there is clear variability in practice both in terms of frequency and method of follow-up imaging. About 59% favored indefinite, life-long follow-up for small unruptured intracranial aneurysms, and favored non-contrast MR angiography for aneurysm follow-up (5).

This heterogeneity and variability in practice necessitate tools and models that can improve overall recommendations. More recently AI has started to gain attention from researchers and clinicians alike for this problem. Numerous researchers have been exploring artificial intelligence (AI) and Deep Learning's (DL) ability in the field of aneurysm detection, rupture prediction, and also improvement of workflow. AI can perform tasks such as pattern recognition, object identification, and problem resolution with human-like intelligence. Based on the data collected for training, AI can make autonomous decisions. Similarly, in medicine, AI can recognize a likely diagnosis and select a suitable treatment based on health records or imaging data without any explicit programming. Machine learning (ML) endows AI to learn and train models to extract and memorize features and related parameters. There are three types of ML: supervised (training with specific labels or annotations), unsupervised (training without specific labels and the algorithm clusters data to reveal underlying patterns), and semi- or weakly supervised learning (training with both labeled and unlabeled data to reduce the annotation burden) (4).

Nowadays, deep learning (DL) has shown significant potential in accurately detecting lesions on medical imaging and has reached, or perhaps surpassed, an expert-level of diagnosis (6). DL is a machine learning technique that directly learns the most predictive features from a large data set of labeled images (6). A deeper discussion on deep learning is beyond the scope of this mini-review. Many introductions are written on artificial intelligence; we recommend reading “High-performance medicine: the convergence of human and artificial intelligence” by Topol et al. (7). We also recommend Liu et al. (8) guide to read articles that use machine learning. Briefly, the technique primarily used in neuroimaging is convolutional neural networks (CNN), a subset of Deep Learning. The visual cortex inspires the algorithms based on CNN's which closely resemble human neurons. They are used extensively in image feature selection, classification, and segmentation, etc., (9).

## AI in Intracranial Aneurysm Detection

CT-Angiography and MR-Angiography have been used widely to screen for IAs. Manual detection of pathologies from images is a laborious process and requires 3D modeling to accurately depict vessel morphology. The process is time-consuming and slow. With the advent of computer vision and deep learning, we can have AI directly analyze images for the presence of IAs. However, with newer imaging techniques and newer models, we gradually saw the AI performance increase compared to human counterparts and at times surpass it. This directly improves the detection rate. As earlier detection allows closer monitoring and eventually results in better patient selection for intervention.

In 2020, Zhao et al. (10) compared the performance of their 3D CNN segmentation model DAResUNET trained on 1,117 Digital Subtraction Angiography (DSA) images against six board-certified radiologists and two neurosurgeons. For aneurysms > 5 mm in size, the ML model had a superior accuracy of 100%. However, four aneurysms < 3 mm in size were missed resulting in a sensitivity of 98.6% for aneurysms < 3 mm in size (10).

Aneurysms can be missed due to the small size and complexity of intracranial vessels. AI can be used to overcome this complexity; as shown by Yuki et al. In their case series, their AI model ResNet-18 detected 5 aneurysms < 2 mm in size by using maximum intensity projection (MIP), to analyze the image in different rotational views. Unfortunately, two board-certified radiologists initially had missed the same five aneurysms. While a human operator can analyze different images, it is time-consuming. ML models can assist in improving accuracy as now we have higher computational power, thus decreasing the latency in detection as these models can predict in near real-time (11).

This was rightly demonstrated by Yang et al. CNN-based algorithm that detected cerebral aneurysms with a sensitivity of 97.5% among 649 computational tomography (CT) images. It also detected eight new aneurysms that were missed in the initial reports, thereby improving the overall performance of radiologists in terms of area under weighted alternative free-response receiver operating characteristic from 0.60 to 0.61. The study showed that AI has good potential when it comes to being a supportive tool for radiologists rather than a replacement (12).

This potential was further explored by Faron et al. (13) who compared the performance of a DEEPMedic CNN model to detect aneurysms from clinical TOF-MRA data vs. two expert human readers. No statistically significant difference was found in the overall sensitivity (OS), showing that AI has now started to reach human-level accuracy. However, when the human reader and CNN's detections were combined, the OS of both human readers was improved (reader 1: 98 vs. 95%, *P* = 0.280; reader 2: 97 vs. 94%, *P* = 0.333). In fact, four previously undetected aneurysms were detected with this combination, reinforcing the fact that AI will continue to improve our diagnostic capabilities and definitely improve patient outcomes (13).

## AI in Intracranial Aneurysm Screening

The burden of disability and mortality from UIAs is significant, while routine screening is challenging because of financial resources, logistical resources, contrast, and radiation load. Moreover, the growth of aneurysms can be non-linear, so the timing of follow-up scans needs to be further personalized. Health insurance-related implications can also arise due to the detection of an IA. We need better guidelines for targeted screening of high-risk individuals and more sophisticated tools to time follow-up studies. These need to be evidence-based tools. AI-based increase in detection of UIAs will help us improve detection on individual patients and improve our detection strategy at a population level.

AI and ML can help us better identify screening targets. Current UIA screening guidelines in the United States and Korea are limited to two categories: (1) patients with at least two family members with UIA or SAH, and (2) patients with a history of autosomal dominant polycystic kidney disease (ADPKD), coarctation of the aorta, or microcephalic osteodysplastic primordial dwarfism. Heo et al. (14) extracted data from the National Health Screening Program in Korea containing general health examinations from 2009 to 2013. Using 21 variables from this data, Logistic Regression (LR), Random Forest (RF), eXtreme Gradient Boosting (XGB), and Deep Neural Network (DNN) were trained, among which the highest area under receiver operating curve (AUROC) value was achieved by the XGB algorithm (0.765) (95% CI 0.742–0.788). The authors stratified the risk group into five categories. This risk stratification with the help of AI models will help in improved targets for screening. This targeted screening in the future with the use of multimodal data: health status & history, family and similar population imaging analysis, genetics will eventually create new guidelines for us to follow (14).

## AI in Intracranial Aneurysm Rupture Prediction

Rupture prediction is the holy grail of prediction modeling. AI can significantly improve rupture prediction, saving lives and limbs in the process. Conventional logistic regression (LR) has been one of the most studied statistical models to predict the rupture status of unruptured intracranial aneurysms (UIAs). In recent years, numerous studies have been done to develop and compare the utility of ML in the prediction of aneurysm rupture risk in the context of statistical models.

Multimodal data is needed for ML to improve its accuracy. Data only limited to imaging does not perform as well in prediction modeling. Chen et al. concluded that integrating clinical, aneurysm morphological, and hemodynamic parameters improves rupture prediction (15).

Similarly, Detmer et al. also compared the logistic regression probability model (LRM) to other ML classifiers by training both the models with hemodynamic, morphological, and patient-related information of 1,631 intracranial aneurysms. The predictive performance of ML classifiers was comparable to the group lasso model. They concluded that incorporating additional information such as aneurysm vessel wall enhancement would lead to better performance of the ML classifiers (16).

We have used scores in clinical practice to predict rupture—one of the widely used includes the PHASES Score (17). Zhu et al. compared ML models with statistical methods and PHASES score in intracranial aneurysm stability assessment. Among the authors' three ML models [Artificial Neural Network (ANN), RF and Support Vector Machine (SVM)], ANN showed the best performance with an area under the curve (AUC) of 0.851 (95% CI 0.828–0.873). Interestingly, even the least performing ML model, RF (0.832 (95% CI 0.809–0.855) significantly outperformed the statistical models and the PHASES score (*P* = 0.045 and *P* < 0.001, respectively). Thus implying that ML models provide better accuracy when compared to commonly used statistical tools such as logistic regression (LR). The superiority of ML over traditional statistical methods can be attributed to the fact that ML has the capacity to simultaneously process massive numbers of variables and can model non-linear relationships while LR and PHASES are limited to linear relationships (18).

## AI in Intracranial Aneurysm in Clinical Decision Support

Therapeutic planning after detection of UIA is very complex and depends on patient factors as well as the characteristics of the aneurysm. Regarding the aneurysm, its location, size, and feeding artery are a few parameters. Meanwhile, a neurovascular multidisciplinary team must be involved to analyze the risk of aneurysmal rupture, the risks of endovascular and surgical treatment, and the predicted outcome of treatment taking into account factors such as the patient's age, lifestyle, comorbid conditions, and personal preferences (19).

Thus AI models that can analyze multiple parameters simultaneously and work with large volumes of data can aid this complex decision making. In addition, AI can enhance the process by adding additional objective data of flow and morphological characteristics of aneurysms. This will lead to an improved occlusion rate of aneurysms and potentially decreased recanalization rates.

In 2019, Bhurwani et al. (20) developed a DNN using Keras to predict occlusion treatment outcomes as a binary output: occluded or unoccluded using only intraoperative information. They analyzed 190 CAs pre and post. This feasibility study concluded with quantitative imaging information that is normalized and improves prediction. Also, shown parameters at an individual level can improve accuracy (20).

As previously mentioned, we have used statistical modeling to develop clinical scores to predict occlusion rates. One such score DIANES score is being used. To predict occlusion success, they used six features (IA diameter, indication, parent artery diameter ratio, neck ratio, side-branch artery, and sex) (21). Williams et al. (22) developed Aneurysm Occlusion Assistant (AnOA), a platform approach is envisioned to assist in real-time decision making. They developed models that can more reliably help with therapeutic decision-making. They have envisioned a new platform that would be able to bring the analytical frameworks from the lab into clinical settings to guide real-time decision making; the Aneurysm Occlusion Assistant (AnOA), an AI assistant based on Keras, Tensorflow, and skLearn that aims to assist neurosurgeons intra-operationally in order to personalize endovascular aneurysm treatments better. Although this has not been tested clinically, the system used pre-and post-device placement data as input and allowed for segmentation of IAs and cranial vasculature with a dice index of ~0.78 and was able to predict aneurysm occlusion at 6 months with accuracy 0.84, in 6.88 (22).

This exemplifies the future of AI in clinical decision-making, and these platforms will start integrating as clinical evidence is accumulated. We will see similar approaches being applied to therapeutic planning as we have already seen in the field of cardiology (23, 24). In the future, we will see these models tested in real-world scenarios and give real-time automatic suggestions for therapeutic planning.

## Documentation, Quality, and Workflow Enhancement Using AI

There is a significant shortage of neuroradiologists, especially in the developing world. In addition, there is an increasing burden of newer forms of imaging data, including 2D and 3D imaging, even in the developed world. AI can augment radiologists in their workflow by decreasing latency, improving accuracy, and automatically performing repetitive tasks like measurements to save time. Dai et al. used modified 2D CTA images produced by the nearby projection (NP) method to train a fast Region-Based Convolutional Neural Network (RCNN) model. The model automatically proposed rectangular areas on the images that may contain aneurysms so that radiologists can easily find them and check whether these proposals were correct or not. The model was 91.8% sensitive in detecting aneurysms automatically. While an experienced radiologist takes around 10 to 15 min to complete one aneurysm image diagnosis, the authors discuss that with their model, it will only take a few minutes for a radiologist to observe all the proposals so that they can save almost 10 min per case, thereby improving efficiency and significantly reducing detection time.

One of the more explored aspects of AI in improving workflow has to be automated note creation using speech recognition software. This has allowed physicians to save time as machine learning converts speech into text (25). An intelligent Electronic Medical Record (EMR) can generate an automated summary resulting in a timely wrap-up of the care process. In addition, AI algorithms can be integrated into various steps in patient management, thus streamlining this critical process. Recently, Williams et al. have reported one of the first papers discussing Aneurysm Occlusion Assistant (AnOA), a semi-autonomous system that uses AI, which can predict the surgical outcome of an IA immediately following device placement, allowing for therapy adjustment. This study marks a crucial step toward incorporating machine learning and data-driven algorithms into surgical suites for better treatment outcomes.

## Challenges and Limitations of AI in Intracranial Aneurysm

Despite significant advancements, this technology is still in its infancy, and before deploying it in a clinical setting, it needs to be thoroughly tested. Given that the majority of data will be imaging-based, we will need faster bandwidth and more processing with dedicated hardware built from the ground up dedicated to processing AI-based tasks. Newer computer chips are now aggressively integrating AI at the level of chip design (26).

The continued biggest challenge in non-imaging data (namely EHR-based data) remains a considerable bottleneck in innovation in healthcare in general and AI in particular. We need more and better standardization of data, improved data sharing, and API-based integrations. The new Cures act has addressed a few of these issues, but we still have a long way to go (27, 28).

## Conclusion

Recent evidence shows that AI, especially Deep Learning, is evolving as a promising aide in clinical decision making in medicine. AI grants us the computational power to explore complicated non-linear relationships in extensive amounts of data, and its predictive power increases with the available datasets for training. Thus the massive amount of data accumulating in clinics, hospitals and stored in electronic medical records through standard tests and medical imaging allows for more applications of AI and high-performance data-driven medicine. With the need for well-trained radiologists and the amount of imaging data generated in healthcare settings worldwide, AI-based CADs will be a tool that will help neuroradiologists streamline clinical workflow while approaching clinical problem solving efficiently and accurately. Our review explored the frontier on how AI can detect aneurysms, evaluate rupture risk, help in triaging clinical therapy strategies, predict treatment outcomes and enhance workflow. Although we have not quite yet reached the threshold for routine clinical application, we believe that with the availability of larger datasets, AI has great potential to solve intracranial aneurysm management issues in a patient-centric manner. Evidence suggests that AI models have started to match and even outperformed human readers on numerous occasions while interpreting medical images. Thus, it would not be an understatement to say that an AI-powered real-time decision-making assistant software for clinics, hospitals, and operating suites will be a norm in the coming years. Artificial Intelligence in neuroradiology; the future is already here.

## A Glimpse into the Future-AI-Enhanced Intracranial Aneurysm Care

A 24-year-old female had a right-sided pounding headache with left-sided weakness, numbness, and tingling. She was rushed to the ER with concern for acute ischemic stroke however her final diagnosis was hemiplegic migraine. A CTA was done in accordance with the standard of care for acute stroke. The neurologist focused on stroke and then migraine. However, a 4 mm aneurysm was also detected in her internal carotid artery with the help of an AI-based system and recommended intervention. The system had evaluated patient rupture risk not just on the size as previously thought to be the main criteria. Rather using multimodal data including imaging; feeding artery, diameters, ratios, radiomic; flow mechanics, genomics, and metabolomics. On the day of the procedure, an AI-based software analyzed real-time images of digital subtraction angiography (DSA) and recommended the type and size of the stent to be used. Post-procedure the appropriate choice of antithrombotics was assisted by AI according to information of her genomics on file. This added case went to a central registry which helped identify two of her relatives for screening. One of whom underwent an aneurysm occlusion procedure for her undiagnosed 8 mm aneurysm.

## Author Contributions

AM: did initial systemic search to find articles, then proceeded to with progressive summarization, and authored majority of the article with JK. AS: did initial systemic search to find articles and then proceeded with progressive summarization. SP: independent search and helped with current and future clinical relevance of said articles. OZ: reviewed and summarized articles for accuracy and relevance. JK: contributed in all aspects of the manuscript including editing and writing the final manuscript. All authors contributed to the article and approved the submitted version.

## Conflict of Interest

JK is employed by VeeOne Health. The remaining authors declare that the research was conducted in the absence of any commercial or financial relationships that could be construed as a potential conflict of interest.

## Publisher's Note

All claims expressed in this article are solely those of the authors and do not necessarily represent those of their affiliated organizations, or those of the publisher, the editors and the reviewers. Any product that may be evaluated in this article, or claim that may be made by its manufacturer, is not guaranteed or endorsed by the publisher.
